# Supplementary material for: Genetic variation as a long-distance modulator of RAD21 expression in humans
Source: Sci Rep. 2022 Jul 29;12:13035. doi: 10.1038/s41598-022-15081-1 (PMC9338076; doi:10.1038/s41598-022-15081-1)
Supplement: Supplementary file 2 — Supplementary Legends. [file 41598_2022_15081_MOESM2_ESM.pdf]

Tables

Supplementary Table 1. A genome-wide search of all 42,953,834 SNPs in dbSNP151 revealed 123 SNPs with significant (FDR < 0.05) regulation of *RAD21* transcript levels. These results were attained through spatial regulatory connections (Hi-C libraries of various tissue origins) confirmed by expression Quantitative Trail Locus (eQTL) analysis (in each tissue available in the GTEx database).

Supplementary Table 2. All SNPs with significant SNP-*RAD21* spatial-eQTL relationships tested for other genes they co-regulate.

Supplementary Table 3. All genes were annotated for significant biological and functional enrichment using g:Profiler, which includes the Kyoto Encyclopedia of Genes and Genomes (KEGG) Pathway Database for pathways and TRANSFAC for transcription factor binding enrichment.

Supplementary Table 4. All genes which comprise the mitotic cohesin and support its loading and unloading, along with three of the seven *RAD21*-co-regulated genes are LoF-intolerant (pLI < 0.9). We also report the 90% upper bound of the loss-of-function confidence interval (LOEUF).
